# Universal terminal for cloud quantum computing

**DOI:** 10.1038/s41598-024-65899-0

**Published:** 2024-07-04

**Authors:** Mohammadsadegh Khazali

**Affiliations:** Department of Physics, University of Tehran, 14395-547 Tehran, Iran

**Keywords:** Quantum information, Atomic and molecular interactions with photons

## Abstract

To bring the quantum computing capacities to the personal edge devices, the optimum approach is to have simple non-error-corrected personal devices that offload the computational tasks to scalable quantum computers via edge servers with cryogenic components and fault-tolerant schemes. Hence the network elements deploy different encoding protocols. This article proposes quantum terminals that are compatible with different encoding protocols; paving the way for realizing mobile edge-quantum computing. By accommodating the atomic lattice processor inside a cavity, the entangling mechanism is provided by the Rydberg cavity-QED technology. The auxiliary atom, responsible for photon emission, senses the logical qubit state via the long-range Rydberg interaction. In other words, the state of logical qubit determines the interaction-induced level-shift at the central atom and hence derives the system over distinguished eigenstates, featuring photon emission at the early or late times controlled by quantum interference. Applying an entanglement-swapping gate on two emitted photons would make the far-separated logical qubits entangled regardless of their encoding protocols. The proposed scheme provides a universal photonic interface for clustering the processors and connecting them with the quantum memories and quantum cloud compatible with different encoding formats.

## Introduction

Complex quantum algorithms demand large-scale fault-tolerant quantum processors. However, current noisy devices encounter error scaling issues as qubit numbers increase, hindering the execution of intricate tasks. Error correction becomes imperative, achieved through logical qubit encoding across multiple physical qubits protected by error-correction codes^1,2^. Quantum operations at the logical level necessitate an abundance of operations at the physical qubit level, entailing costly techniques like complex optimization^3,4^, magic state distillation^5^, transversal gates^1^, and lattice surgery^6–8^. Consequently, the computational power and capacity requirements for quantum processors skyrocket. Moreover, accessibility to fault-tolerant quantum computation on personal devices remains constrained by the need for laboratory equipment such as laser cooling and cryogenic environments.

To democratize the quantum advantage, an optimized cost-benefit strategy entails the deployment of small-scale, non-error-corrected mobile devices^9^, which delegate computational tasks to error-corrected servers^10^. Consequently, elements within the quantum cloud necessitate different encoding protocols. A plug-and-play approach for clustering and connecting these devices demands universal terminals compatible with all encoding schemes.

This paper introduces a novel scheme designed to entangle a single photonic qubit with a logical qubit encoded across 4 atoms^11^.

1 $$\begin{aligned} {|{0}\rangle }_L=\frac{{|{0000}\rangle }+{|{1111}\rangle }}{\sqrt{2}}, \quad \, {|{1}\rangle }_L=\frac{{|{0101}\rangle }+{|{1010}\rangle }}{\sqrt{2}}. \end{aligned}$$

Additionally, the extension to alternative encoding protocols, such as 6-qubit^12^ and 13-qubit^13^, is explored. By employing a projective Bell-state measurement (PBM) on two flying qubits, logical qubits in stationary units become entangled regardless of their encoding scheme. This universal photonic interface holds significant value for clustering fault-tolerant processors^14^, connecting them with quantum memories^14–16^, and integrating them within the quantum cloud infrastructure^10^.

**Figure 1:**
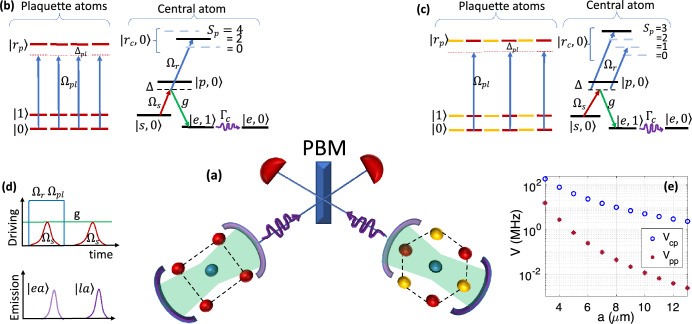
Universal terminal for long-distance entanglement of logical qubits with different encoding formats. (**a**) The auxiliary atom at the center of the plaquette emits a photon at the early or late time, conditioned on the state of logical-qubit encoded on plaquette atoms. Subsequent PBM of photons entangles logically distant qubits. (**b**, **c**) Level scheme of the operation in (**b**) 4-qubit and (**c**) 6-qubit encoding schemes shown in the right and left cavities of (**a**). The lambda configuration for the central atom involves atom-laser $\Omega _s$ and atom-cavity *g* couplings, facilitating photon emission. To discern the logical qubit, partial Rydberg population on both the plaquette and central atom is essential. As a result, the interaction-induced level-shift of the central atom’s Rydberg state depends on the plaquette’s spin-state $S_p$. In (**b**) the $|0_L\rangle$ logical state is associated with plaquette spin state $S_p=0$ and 4, making $\Omega _r$ laser out of resonance with the Rydberg level. Hence, the Raman transition from $|s\rangle$ to $|e\rangle$ would generate a single photon at the $|\text {early}\rangle$ time. Conversely, the $|1_L\rangle$ logical state associates with $S_p=2$, aligning the $\Omega _r$ laser in-resonance with the Rydberg level. Consequently, destructive interference inhibits the transition to $|e\rangle$, blocking early photon emission. The subsequent exclusive $\Omega _s$ pulse, depicted in the timeline diagram (**d**), triggers $|\text {late}\rangle$ photon emission in the case of $|1_L\rangle$ state. (**e**) The chosen Rydberg states in *Cs* are $|r_p\rangle =|50p_{3/2},1/2\rangle$ and $|r_c\rangle =|50s_{1/2},1/2\rangle$, creating a distinction between inter- ($V_{cp}$) and intra-component ($V_{pp}$) interactions at large interatomic distances.

A logical qubit represents a highly entangled two-dimensional subspace within the broader Hilbert space of multiple physical qubits. To circumvent the expensive techniques associated with logical operations^1,3–8^, leveraging system-specific properties can substantially reduce the number of operations performed on physical qubits and consequently minimize errors during execution. Laser-excited Rydberg atoms emerge as a promising platform for implementing surface codes^13,17,18^, with their long-range interactions enabling simultaneous operations on multiple qubits^19–24^, and offering additional opportunities in quantum optics^25–35^.

Considering atomic-lattice processors embedded within cavities^34–41^, this article proposes a universal terminal to connect widely separated units utilizing different encoding schemes. The proposed scheme employs cavity-QED techniques to emit an early or late photon from an auxiliary central atom, conditioned on the logical qubit encoded within the surrounding plaquette atoms. Different states of the logical qubit induce distinct interaction-induced level-shifts on the central atom’s Rydberg state, facilitated by either the Rydberg-dipolar or Rydberg–Fermi interaction. Consequently, the logical qubit dictates which eigenstate the central atom follows, either involving or excluding the atom-cavity coupling. Consequently, the logical qubit becomes entangled with the time-bin photonic qubit emitted by the central atom, yielding the entangled state.

2 $$\begin{aligned} ({|{\text {early}}\rangle }{|{\text {0}_L}\rangle }+{|{\text {late}}\rangle }{|{\text {1}_L}\rangle })/\sqrt{2}. \end{aligned}$$

Subsequent PBM on two photons in the time-bin basis^42–48^ entangles the stationary qubits regardless of their encoding schemes. The time-bin qubit emerges as an ideal choice due to its inherent robustness against phase fluctuations^42^, with recent advancements in achieving high-fidelity operations^49^.

By leveraging system-specific properties, the current scheme operates on all physical qubits in a single step, circumventing the costly techniques typically required for logical operations^1,3–8^. In the Dipolar scheme, both the physical and central atoms are excited to the Rydberg level via simultaneous pulses. In contrast, the alternative Rydberg–Fermi approach involves a single pulse exclusively applied to the central atom, operating via Fermi scattering^50–52^ of the central atom’s Rydberg electron from the plaquette atoms in a spin-dependent lattice^53–64^. Simulations of the entangling operation under major decoherence sources estimate a high fidelity operation of approximately 99.7% in both schemes.

## Results

### scheme

The setup consists of ^133^Cs atomic lattice quantum processor accommodated in a cavity. Fig. 1a provides a simplified illustration featuring a single plaquette and a central atom within each edge device. Logical information in two devices is encoded using distinct 4- and 6-qubit bases. The system atoms are placed on a square (hexagonal) plaquette consisting of physical qubit states ${|{0}\rangle }={|{6S,F=3}\rangle }$ and ${|{1}\rangle }={|{6S,F=4}\rangle }$. The auxiliary atom responsible for conditional photon emission is at the center of the plaquette with the electronic level scheme presented in Fig. 1b,c for the encoding cases.

The level scheme of the central atom features a lower lambda configuration responsible for on-demand single-photon emission^65–67^. The $\Lambda$ transition can be allowed or prohibited based on quantum interference, contingent upon the off-resonance or in-resonance status of the upper laser $\Omega _r$ with the Rydberg transition^68^. The tuning of the $\Omega _r$ laser is dictated by the logical qubit through dipolar interaction. This long-distance sensing is facilitated by Rydberg dressing of plaquette atoms in the ${|{0}\rangle }$ qubit state, exciting partial Rydberg population $P_r=w_d^2=(\Omega _{pl}/2\Delta _{pl})^2$ on each plaquette atom in ${|{0}\rangle }$ state, with $\Omega _{pl}$ and $\Delta _{pl}$ denoting the Rabi frequency and detuning of the exciting laser for plaquette atoms.

In the lower lambda system, the transition between states ${|{s}\rangle }={|{6S_{1/2}, F=4,m_F=4}\rangle }$ and ${|{p}\rangle }={|{7P_{3/2},F=4,m_F=4}\rangle }$ is induced by the $\Omega _s$ laser. The transition from ${|{p}\rangle }$ to ${|{e}\rangle }={|{6S_{1/2}, F=3, m_F=3}\rangle }$ is governed by the Jaynes-Cummings interaction with coupling constant *g* and cavity mode frequency $\omega _c$. These transitions are detuned from the intermediate state by $\Delta$. The state basis ${|{i,n}\rangle }$ in Fig. 1b,c encompasses the electronic state ${|{i}\rangle }$ of the central atom and the cavity number state ${|{n}\rangle }$. Over a Raman $\pi$ pulse, the transition from ${|{s,0}\rangle }$ to ${|{e,1}\rangle }$ would generate a photon in the cavity. Decay of the cavity mode leads to single-photon emission, and the system stabilizes in the state ${|{e,0}\rangle }$.

Dressing the plaquette atoms in ${|{0}\rangle }$ state with Rydberg level results in an interaction-induced level-shift on the central atom’s ${|{r_c}\rangle }$ state, which is quantified by the plaquette spin $S_p$, see Fig. 1b and c. The plaquette spin is defined as $S_p=\sum _{j\in p} \sigma _{00}^{(j)}$, where $\sigma _{00}={|{0}\rangle }{\langle {0}|}$ represents the projective operator, and *j* spans over the plaquette atoms. In *four-qubit encoding*, having logical qubit ${|{0}\rangle }_L=({|{0000}\rangle }+{|{1111}\rangle })/\sqrt{2}$, the $\Omega _r$ laser would be out of resonance with the interaction-shifted Rydberg level. Consequently, the initial $\Omega _s$ pulse in Fig.1d elicits an early photon emission, denoted as ${|{\text {early}}\rangle }$.

Conversely, for the alternative logical qubit ${|{1}\rangle }_L=({|{0101}\rangle }+{|{1010}\rangle })/\sqrt{2}$, two plaquette atoms are dressed by the Rydberg state since $S_p=2$. Consequently, the interaction-induced level-shift aligns the $\Omega _r$ laser in-resonance with the Rydberg level, blocking photon emission during the first $\Omega _s$ pulse. Subsequently, the preserved population in ${|{s,0}\rangle }$ would now get transferred over the second $\Omega _s$ pulse, unaccompanied by the Rydberg lasers $\Omega _r$, and hence emits a late photon ${|{\text {late}}\rangle }$. This sequence leads to the desired entangled state described in Eq. (2). The temporal arrangement of pulses is illustrated in Fig. 1d, and the underlying physics governing blocking and transmission are further elaborated in Section “Entangling operation”.

A similar mechanism can be extended to *six-qubit*^12^ or *thirteen-qubit*^13^ encoding schemes, where the logical basis comprises odd and even parity states of the $\bar{Z}=I Z I Z I Z$^12^ or $\bar{Z}=IIII Z I Z I Z IIII$^13^ logical operators with weight three. By situating three physical qubits with *Z* Pauli matrices around the photon-emitting atom, the central auxiliary atom can detect and entangle the state of the logical qubit with the time-bin qubit. With recent advances in moving atoms in Rydberg processors^13^, the proposed scheme can be tailored to any form of logical encoding. In Fig. 1c, dressing the ${|{0}\rangle }$ qubit state of the three targeted sites with Rydberg levels induces an effective level-shift on the central atom, quantified by the spin-number of the three plaquette atoms.

Within the level scheme of the central atom, the two-color $\Omega _r$ lasers (Fig. 1c) are tuned in resonance with the odd parity of the $\bar{Z}$ stabilizer. The generation of these two-color $\Omega _r$ transitions can be achieved using a single laser in a setup involving beamsplitters and acousto-optical modulators. For even parity of $\bar{Z}$, the $\Omega _r$ laser would be out of resonance with the Rydberg level. Hence, the initial $\Omega _s$ pulse in Fig. 1d triggers early photon emission ${|{\text {early}}\rangle }$. However, with an odd number of spin-downs in the three targeted atoms, one of the $\Omega _r$ lasers aligns with the Rydberg level resonance, while the other induces a level-shift to the ${|{p}\rangle }$ state, resulting in a slight correction in the detuning value $\Delta \equiv \Delta \pm \Omega _r^2/4w_d^2V_{\text {cp}}$. The resonant laser blocks the transition during the first $\Omega _s$ pulse, as discussed in Section “Entangling operation”. Subsequently, the preserved population in ${|{s,0}\rangle }$ transfers over the second $\Omega _s$ pulse (unaccompanied by $\Omega _r$), leading to the emission of a late photon ${|{\text {late}}\rangle }$, yielding the desired state described in Eq. (2) for the 6-qubit encoding. In an entanglement swapping station, photons emitted from distinct sources undergo a C-NOT gate followed by a projective Bell state measurement. This procedure entangles the two far-separated plaquettes in their respective logical bases, as discussed in Section “Entanglement swapping”.

### Entangling operation

This section delves into the physics underpinning conditional photon emission, which entangles the stationary logical and flying qubits. In the case of four-qubit encoding, the ${|{1_L}\rangle }$ state as per Eq. (1) corresponds to two out of four plaquette atoms being in the ${|{0}\rangle }$ state $(S_p=2)$, which gets dressed with Rydberg levels. This leads to an interaction-induced shift of $2w_d^2V_{\text {cp}}$ on the ${|{r_c}\rangle }$ state, aligning the $\Omega _r$ laser in resonance with the Rydberg level ($\delta _{\text {r}}({|{1_L}\rangle })=0$). Conversely, for ${|{0_L}\rangle }$, the plaquette’s configuration involves either $S_p=0$ or 4 atoms dressed to the Rydberg state, resulting in an effective detuning of $\delta _{\text {r}}({|{0_L}\rangle })=2w_d^2V_{\text {cp}}$. The Hamiltonian of the central atom in the electronic basis is given by

3 $$\begin{aligned} H= & \, \Omega _s/2(\hat{\sigma }_{sp}+\text {h.c.}) + \Omega _r/2(\hat{\sigma }_{rp}+\text {h.c.}) \nonumber \\{} & {} + g/2(\hat{\sigma }_{ep}+\text {h.c.})+{\Delta }\hat{\sigma }_{pp}+\delta _{r}\hat{\sigma }_{rr} \quad \quad \quad \end{aligned}$$

where $\hat{\sigma }_{\alpha \beta }={|{\alpha }\rangle }{\langle {\beta }|}$. Following Muller et al^69^ the analytic discussion is simplified by considering the large detuning regime $\Delta \gg \Omega _{\{s,r\}},g$ and equal couplings $g=\Omega _s$.

After adiabatic elimination of the intermediate state ${|{p}\rangle }$, the Hamiltonian is represented in new basis ${|{\pm }\rangle }=({|{s}\rangle }\pm {|{e}\rangle })/\sqrt{2}$, and ${|{r}\rangle }$ as

4 $$\begin{aligned} H/\epsilon =\lambda ^2 {|{+}\rangle }{\langle {+}|} + (1-\delta ) {|{r}\rangle }{\langle {r}|} + \lambda ({|{+}\rangle }{\langle {r}|}+\text {h.c.})\quad \quad \quad \end{aligned}$$

where $\lambda =\Omega _s/\Omega _r$ is the dimensionless Rabi frequency and $\delta =\delta _{r}/\epsilon$ is the interaction induced level-shift scaled by $\epsilon =\Omega _r^2/4\Delta$. In the regime of $\lambda ,\delta \ll 1$ the lower bright state ${|{b}\rangle }=[(1-\delta ){|{+}\rangle }-\lambda {|{r}\rangle }]/\sqrt{(1-\delta )^2+\lambda ^2}$, with the energy $2\delta \lambda ^2$ would interfere with the dark state ${|{d}\rangle }={|{-}\rangle }$.

**Figure 2:**
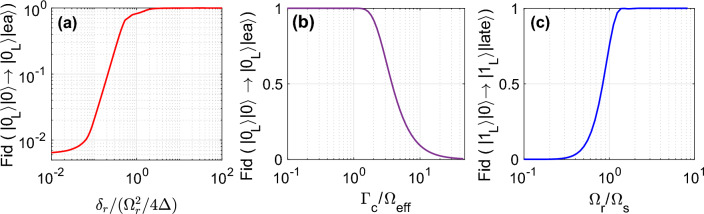
The operation regime of the entangling scheme. The operation is simulated under the non-hermitian Hamiltonian $\tilde{H}$ of Eq. 6. (**a**) With $|0_L\rangle$, the interaction-induced detuning $\delta \gtrsim 1$ lifts the interference of dark and bright states, allowing early photon emission. (**b**) With $|0_L\rangle$ logical state ($\delta _{r}=2w_d^2V_{\text {cp}}$), the optimum early emission occurs at $\Gamma _c\le \Omega _{\text {eff}}$. Larger cavity leakage would reduce the coherence, scatter the population out of the dark state, and reduce the emission probability. (**c**) Having $|1_L\rangle$ logical state ($\delta _{r}=0$), the early photon emission would be blocked for $\Omega _r/\Omega _s>1.5$ by more than 99%. Applied parameters are $\delta _{r}/2\pi =1$MHz, $\Delta /2\pi =390$MHz, $\Omega _r/2\pi =32$MHz, $\max (\Omega _s)/2\pi =10.5$MHz, $g/2\pi =8.5$MHz, $\Gamma =\Omega _{\text {eff}}$, $\sigma \Gamma =2.5$.

Having ${|{1_L}\rangle }$, the Rydberg laser would be in resonance $\delta =0$. Hence, the system would follow the new dark state ${|{D}\rangle }=({|{d}\rangle }+{|{b}\rangle })/\sqrt{2}=({|{s}\rangle }-\lambda {|{r}\rangle })/\sqrt{1+\lambda ^2}$ featuring destructive interference that blocks the transition to ${|{e}\rangle }$ state. Conversely, for ${|{0_L}\rangle }$ the interaction-induced detuning of $\delta =\delta _{\text {r}}/(\Omega _r^2/4\Delta )\gtrsim 1$ lifts the degeneracy of the ${|{d}\rangle }$ and ${|{b}\rangle }$ states. As a result, the system exclusively follows the dark state ${|{d}\rangle }$ and emits an early photon, see Fig. 2a.

A large cavity decay rate $\Gamma _c$ enhances the emission rate and narrows the emission probability profile but comes at the cost of reduced coherence. In the regime where $\lambda =\Omega _s/\Omega _r\ll 1$, the steady-state of the master equation can be obtained perturbatively as $\rho =\rho _0+\lambda \rho _1+\lambda ^2 \rho _2$. ﻿The desired transition coherence is given by

5 $$\begin{aligned} \rho _{se}= \frac{\Omega _s g}{4\Delta } \frac{2i \delta _{r}}{\Gamma _c(\Omega _r^2/4\Delta - \delta _{r})}. \end{aligned}$$

Cavity decay rates $\Gamma _c$ larger than the effective transition Rabi frequency $\Omega _{\text {eff}}=\Omega _s g/4\Delta$ reduce the coherence between ${|{s}\rangle }$ and ${|{e}\rangle }$. Since an incoherent superposition of these states does not exclusively project into the dark state ${|{d}\rangle }$, some of the population would transfer to bright states reducing the operation fidelity, see Fig. 2b. Finally, the condition $\lambda =\Omega _s/\Omega _r<1$ is necessary to block the early transmission of photons in the presence of ${|{1_L}\rangle }$ as numerically evaluated in Fig. 2c.

To study the *Photon emission*, Eq. (3) must be modified to the non-hermitian version

6 $$\begin{aligned} \tilde{H}=H-\text {i}\Gamma _c\hat{\sigma }_{ee}, \end{aligned}$$

where $\Gamma _c$ is the cavity leakage rate. The operation under this Hamiltonian is quantified in Fig. 2. In the regime that $\Delta ,\delta _{r}\gg \Omega _{\{r,s\}},g$, the adiabatic elimination of Rydberg and intermediate *p* state would simplify the dynamics to a two-level system with an effective Rabi frequency of $\Omega _{\text {eff}}=\frac{\Omega _s g}{4{\Delta }} (1+\frac{\Omega _r^2}{4{\Delta }\delta _{r}})$ and an effective detuning of $(\frac{\Omega _s^2}{4\Delta }-\frac{g^2}{4\Delta })(1+\frac{\Omega _r^2}{4{\Delta }\delta _{r}})+\text {i}\Gamma _c/2$. Choosing close coupling strengths $\Omega _s\approx g$ to facilitate the desired transition, the photon flux out of the cavity would be given by $\Phi (t)=\Gamma _c|c_{e,1}|^2=\Gamma _c\frac{\Omega _{\text {eff}}^2 }{\tilde{\Omega } ^2} e^{-\Gamma _c t} \sin ^2(\tilde{\Omega } t)$ where $\tilde{\Omega }=\sqrt{\Omega _{\text {eff}}^2+\Gamma _c^2/4}$﻿.

### Entanglement swapping

Entangling far-separated atomic qubits in the logical basis requires Bell state projective measurement on their entangled emitted photons. The proposed Rydberg cQED scheme provides entanglement between the logical qubit and the emitted photon in each cavity setup, see Eq. (2). The state could then be rearranged in separate logical and photonic qubit pairs in the following line

$$\begin{aligned} {|{\psi }\rangle }= & {} \frac{({|{1_L}\rangle }{|{\text {late}}\rangle }+{|{0_L}\rangle }{|{\text {early}}\rangle })_1}{\sqrt{2}}\frac{({|{1_L}\rangle }{|{\text {late}}\rangle }+{|{0_L}\rangle }{|{\text {early}}\rangle })_2}{\sqrt{2}} \nonumber \\= & {} (|{\tilde{\phi }}^{+}_L\rangle |\tilde{\phi }^{+}_p\rangle + |{\tilde{\phi }}^{-}_L\rangle |\tilde{\phi }^{-}_p\rangle +|{\tilde{\psi }}^{+}_L\rangle |\tilde{\psi }^{+}_p\rangle +|{\tilde{\psi }}^{-}_L\rangle |\tilde{\psi }^{-}_p\rangle )/2, \end{aligned}$$

where the rotated Bell states are obtained by applying a Hadamard on the second element i.e. $|\tilde{\phi }^{\pm }\rangle = {|{1}\rangle }_1{|{+}\rangle }_2\pm {|{0}\rangle }_1{|{-}\rangle }_2$ and $|\tilde{\psi }^{\pm }\rangle = {|{0}\rangle }_1{|{+}\rangle }_2\pm {|{1}\rangle }_1{|{-}\rangle }_2$ where $|\pm \rangle =( {|{1}\rangle }\pm |0\rangle )/\sqrt{2}$. The photonic states follow the same presentation format on an early and late basis. Applying a CZ gate on the photonic states^70^ written in the four rotated Bell states would result in

7 $$\begin{aligned} \text {CZ}_p{|{\psi }\rangle }= & {} (|++\rangle _{p} |\tilde{\phi }^{+}\rangle _{L} -|--\rangle _{p} |\tilde{\phi }^{-}\rangle _{L} \nonumber \\{} & {} -|-+\rangle _{p} |\tilde{\psi }^{+}\rangle _{L} - |+-\rangle _{p} |\tilde{\psi }^{-}\rangle _{L} )/2. \end{aligned}$$

Subsequent projective measurement of the photonic pair in the Bell basis^71^ guarantees the entanglement of logical qubits.

### Quantifying the operation fidelity

In sensing the logical qubits, it is important to maximize the interaction of central and plaquette atoms $V_{cp}$ while suppressing the cross-talk among the plaquette sites $V_{pp}$. A typical approach is to choose the inter-component interaction to be in-resonance $V_{cp}\propto n^4/R^3$ and the intra-component to be perturbative $V_{pp}\propto n^{11}/R^6$. The desired parameter to be optimized is then $V_{cp}/V_{pp}=R^3/n^7$ which suggests using relatively small *n* and large interatomic distances. On the other hand, going to large *R* makes the gate slower, and smaller *n* enhances the spontaneous emission from the Rydberg levels $\gamma _r\propto n^{-3}$. The optimal choice of states used for this gate is ${|{r_p}\rangle }={|{50P_{3/2},1/2}\rangle }$ and ${|{r_c}\rangle }={|{50S_{1/2},1/2}\rangle }$ with quantization axis being perpendicular to the lattice plane, see Fig. 1e.

Here, the performance of the scheme for the four-qubit encoding case is studied. Considering a dressing parameter of $w_d^2 = (\Omega _{pl}/2\Delta _{pl})^2 = 0.1$ for the plaquette atoms, and a lattice constant of $a = 4.5\mu$m, the interaction-induced detuning would be $\delta _r/2\pi = 1.7\text {MHz}$. The optimum realistic parameters in driving the central atom include $\Omega _{s0}/2\pi = 10.5\text {MHz}$, $g/2\pi = 8.5\text {MHz}$, $\Omega _r/2\pi = 52\text {MHz}$, $\Gamma _c/2\pi = 114\text {kHz}$^72^, and $\Delta /2\pi = 390\text {MHz}$. The time interval of a Gaussian pulse $\Omega _s(t) = \Omega _{s0}\exp (-t^2/2\sigma ^2)$ must be long enough to fulfill the adiabaticity condition to preserve the dark state, $\sigma \gg g^{-1}$, and to shape the profile of the emitted photon, $\sigma \ge \Gamma ^{-1}$.

The error budget is as follows. Considering the spontaneous emission rates of the intermediate state ($\gamma _{p}/2\pi = 1\text {MHz}$)^73^ and the Rydberg states at cryogenic temperatures (T=77K)^74^, both encountered throughout a Gaussian pulse with $\sigma = 2.4\mu$s and a total duration of 20$\mu$s, the losses from the plaquette dressed states and the central atom over the operation time for an initial logical Bell state would average to 0.02 and 0.0012 respectively.

The unwanted accumulated phase from the inter-component interaction can be calculated as $\sum _{i<j}w_d^4V_{pp}(r_{ij})\tau$, resulting in 0.08 and 0.008 rad over 20$\mu$s for the physical qubit states ${|{1111}\rangle }$ and {${|{1010}\rangle }$, ${|{0101}\rangle }$} respectively. With an initial Bell state $({|{0_L}\rangle } + {|{1_L}\rangle })/\sqrt{2}$, these unwanted phases contribute to an infidelity of 0.0012. In the case of ${|{1_L}\rangle }$, the central atom acquires Rydberg population. However as discussed in the methods, the resonant interaction channel responsible for strong dipolar interaction forms a dark state that does not accumulate any phase. The other non-resonant dipole-coupled Rydberg pairs form a weak perturbative interaction at this interatomic distance, resulting in a small undesired phase accumulation of 0.013 and 0.0068 rad for the physical qubit configurations ${|{1111}\rangle }$ and {${|{1010}\rangle }$, ${|{0101}\rangle }$} respectively over the gate operation. The corresponding error applied to the initial logical Bell state would be 0.00002.

The conditional transfer of population in the central atom from ${|{s}\rangle }$ to ${|{e}\rangle }$ and photon emission is simulated under the Hamiltonian of Eq. (6), resulting in a fidelity of 99.97% with the above-mentioned parameters. However, encountering population leakage to the Rydberg manifold due to resonant dipole coupling, this fidelity reduces to 99.72%, as detailed in the methods section. Considering these errors, the proposed operation ${|{0}\rangle }({|{0_L}\rangle } + {|{1_L}\rangle })/\sqrt{2} \rightarrow ({|{\text {early}}\rangle }{|{0_L}\rangle } + {|{\text {late}}\rangle }{|{1_L}\rangle })/\sqrt{2}$ is expected to be performed with a fidelity of 97.4% in the four-qubit encoded scheme.

## Discussion

This article proposes a hardware architecture and protocol for long-distance entanglement generation between logical qubits with different encoding protocols. This allows the realization of cloud quantum computation for mobile users with simple quantum devices. The proposed photonic quantum bus solution promises the scalability of fault-tolerant processors at the current NISQ-era devices. This would resemble the integrated circuits (IC) technology of silicon-based processors characterized by Moore’s law^75^. With the transition/processing limits of the quantum satellites^76–79^ and mobile edge devices, communication via a single photon is desired. With large communication channel bandwidth, emitted photons from the terminal could be encoded into three-photons GHZ state^80,81^ to correct the bit-flip, while the photon loss could be neglected by post-selection after the PBM. From a fundamental perspective, the entanglement in logical basis facilitates the investigation of phenomena over a wide dimension of state space, while operating on the basis that is growing polynomially.

## Methods

### Dark-state under resonant interaction

The resonant dipolar interaction denoted as $V_{cp}$, between two Rydberg pairs may prompt concerns regarding potential population loss within the Rydberg manifold. This concern is particularly relevant in the case of ${|{1_L}\rangle }$ state, where the central atom resonates and populates the Rydberg state ${|{r_c}\rangle }$ featuring resonant interaction with ${|{r_p}\rangle }$. In this part, we elucidate how the interplay between laser transitions and resonant dipolar coupling engenders a dark state^19,82^. This dark state ensures the retrieval of ground hyperfine states at the end of the pulse. Furthermore, it explains why the system does not accrue phase despite the presence of strong resonant dipolar interaction.

For the concerning state, ${|{1_L}\rangle }$, the resonance of $\Omega _r$ with the Rydberg state decouples the state ${|{e}\rangle }$ due to destructive interference, leaving the two-photon Rydberg exciting transition, see Section “Entangling operation”. Upon adiabatic elimination of the intermediate state ${|{p}\rangle }$, the central atom undergoes laser excitation from ${|{s}\rangle }$ to ${|{r_c}\rangle }$. To present a simplified model, a scenario where a plaquette atom and a central atom are initialized in ${|{r_p}\rangle }$ and ${|{s_c}\rangle }$, is considered. Upon the excitation of the central atom to ${|{r_c}\rangle }$, the dipole resonant coupling denoted as $V_{cp}$ arises between the targeted Rydberg pair ${|{r_cr_p}\rangle }$ and the resonantly dipole-coupled pair ${|{r'_cr'_p}\rangle }$, as elaborated in^19^ regarding the feasible choices of Rydberg states. The effective Hamiltonian governing this process takes the form of an Electromagnetically Induced Transparency (EIT) type:

8 $$\begin{aligned} H_c=\Omega /2 {|{r_pr_c}\rangle }{\langle {r_ps_c}|}+V_{cp}{|{r'_pr'_c}\rangle }{\langle {r_pr_c}|}+h.c. \end{aligned}$$

where $\Omega =\Omega _s\Omega _r/2\Delta$. The eigenstates of this system are represented as follows:

9 $$\begin{aligned}{} & {} {|{\psi _0}\rangle }=(V_{cp} {|{r_ps_c}\rangle }-\Omega /2{|{r'_pr'_c}\rangle }+h.c.)/N \nonumber \\{} & {} {|{\psi _{\pm }}\rangle }=(\Omega /2 {|{r_ps_c}\rangle }\pm \nu {|{r_pr_c}\rangle } + V_{cp} {|{r'_pr'_c}\rangle }+h.c.)/N' \end{aligned}$$

where *N* and $N'$ are the normalization factors, and $\nu =V_{cp}^2+\Omega ^2/4$. The corresponding eigenvalues are $\lambda =0$ and $\lambda _{\pm }=\pm \nu$.

**Figure 3:**
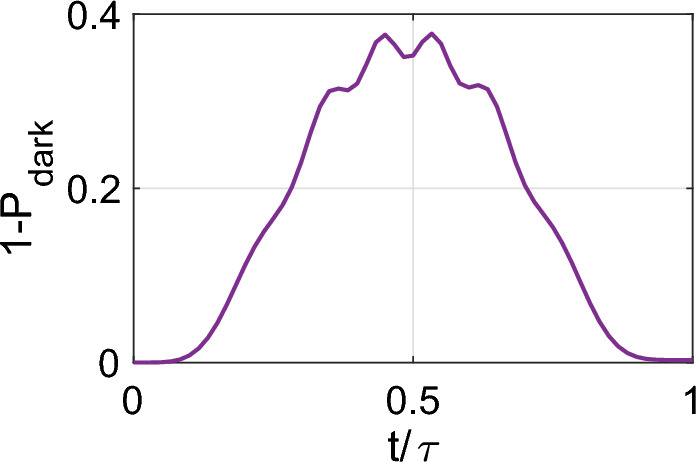
In the case of $|1_L\rangle$, the resonant dipolar interaction $V_{cp}$ combined with the laser excitation of $|r_c\rangle$ forms the dark state described by Eq. (9). During the $\Omega _s=\Omega _{s0}\sin ^2(\pi t/T)$ pulse, some population scatters to bright states but returns by the pulse’s end. This behavior has been explored in the realm of dark state gates^19,82^, ensuring the retrieval of population in the computational basis.

Figure 3 illustrates the evolution of the dark state, beginning from ${|{r_p s_c}\rangle }$ under a sinusoidal $\Omega _s=\Omega _{s0}\sin ^2(\pi t/T)$ pulse with a duration of $T=30\mu s$, with other parameters resembling those in Section “Entanglement swapping”. The change of pulse shape here is due to the sensitivity of the dark state model to the final slope of the pulse. The simulation encompasses states $\{{|{e}\rangle },{|{s}\rangle },{|{p}\rangle },{|{r_c}\rangle },{|{r'_c}\rangle }\}$ and $\{{|{r_p}\rangle }, {|{r'p}\rangle }\}$ for the central and plaquette atoms, respectively. The initial state ${|{r_ps_c}\rangle }$ starts as the dark state ${|{\psi _0}\rangle }$, and in the middle of the pulse it scatters equally to bright states ${|{\psi _{\pm }}\rangle }$, which ultimately returns to the dark state upon termination of the $\Omega _s$ pulse. The deviation from the dark state at the end of the pulse is computed as 0.002.

At this stage, the dark state is pure ${|{r_ps_c}\rangle }$, and no population remains in the coupled pair ${|{r'_pr'_c}\rangle }$ after the operation. Furthermore, since the dark state has zero energy under Eq. (8), the system does not gain any phase from the strong dipolar interaction $V_{cp}$. The scattered population to bright states is symmetrically distributed, and considering the eigenvalues of bright states $\lambda _{\pm }=\pm \nu$, they acquire identical phase values but with opposite signs. As a result, at the end of the $\Omega _s$ pulse, the final state acquires zero phase despite the presence of the strong $V_{cp}$ interaction.

### Implementation via Rydberg–Fermi sensing

**Figure 4:**
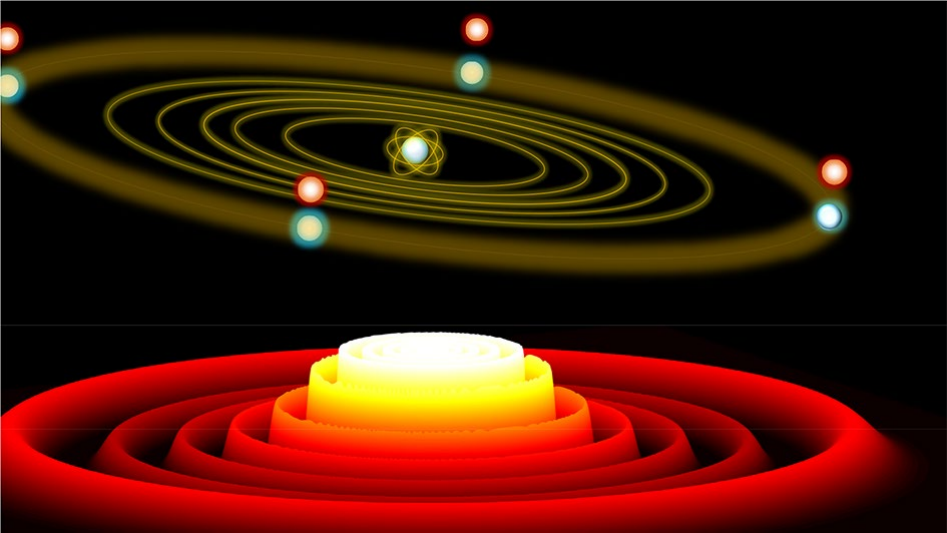
Rydberg–Fermi sensing of the logical qubit: Schematic picture. In a spin-dependent lattice, the plaquette atoms would be in a spatial superposition of being in red and blue sites where the components are controlled by the internal electronic qubit-states $|0\rangle$ and $|1\rangle$. As a result of this dual spin/spatial encoding, only plaquette atoms in $|0\rangle$ state would be accommodated in the Rydberg wave-function and undergo the Fermi scattering. This scattering imposes an interaction-induced level-shift on the Rydberg state that quantifies by the plaquette’s spin-state $S_p$ and controls the photon emission as described in Section “Entangling operation”.

**Figure 5:**
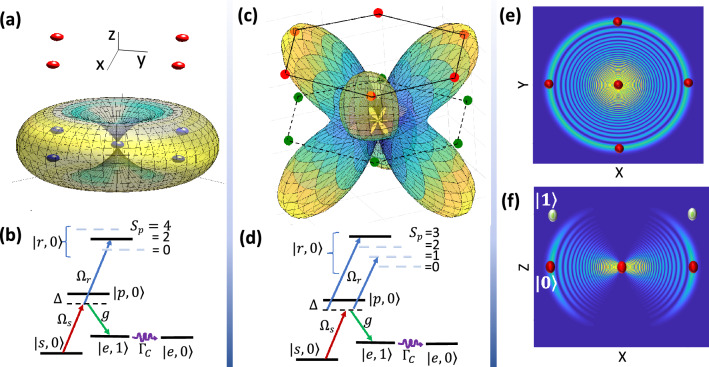
Rydberg–Fermi level scheme: The physical qubits are dual-encoded in the spin/spatial basis of a spin-dependent lattice placed over (**a**) square or (**c**) hexagonal structures in the 4- and 6-qubit encodings, respectively. Exciting Rydberg states with (**a**) $Y_{2}^2(\theta ,\phi )$ and (**c**) $(Y_2^2(\theta ,\phi )+Y_{2}^{-1}(\theta ,\phi ))/\sqrt{2}$ spherical Harmonics results in a Fermi scattering level-shift that distinguishes the logical qubit states. (**b**, **d**) The level scheme is similar to Fig. 1, excluding the plaquette excitation. The energy splitting of the Rydberg state depends on the number of atoms in the Rydberg orbitals $S_p=\sum _i\sigma _{00}^{(i)}$. In (**b**), the $|0_L\rangle$ logical state is associated with $S_p=0$ and 4, making the $\Omega _r$ laser out of resonance and allowing early photon emission. Conversely, $|1_L\rangle$ accommodates two plaquette atoms in Rydberg wave function $S_p=2$, making the $\Omega _r$ laser in-resonance and blocking the early photon emission. (**d**) The two-colored Rydberg excitation $\Omega _r$ get in resonance with the odd parity of $\bar{Z}$, associated with $|1\rangle _L$, thereby blocking the early photon emission. (**e**, **f**) The geometry of th *xy* and *xz* cross-sections of the $|57D_{5/2},5/2\rangle$ electron cloud relative to the position of plaquette atoms in the spin-dependent lattice.

**Figure 6:**
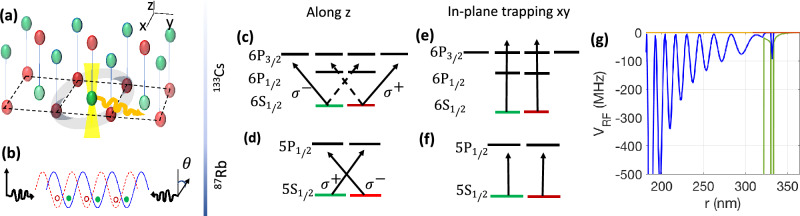
Dual-species spin-dependent lattice. (**a**) In a 2D lattice with a single atom per site, applying a qubit-dependent lattice shift along the $z$-axis places each atom in a spatial superposition of green and red sites, where the components are controlled by the internal electronic qubit states $|0\rangle$ and $|1\rangle$. (**b**) Counter-propagating linearly polarized lights along the *z*-axis with a relative polarization shift of $\theta$ form two standing waves with $\sigma ^{-}$ and $\sigma ^{+}$ circular polarizations. (**c**) The 870 nm trapping laser perpendicular to the lattice plane is aligned between the $6P_{3/2}$ and $6P_{1/2}$ states for Cs, causing the polarizability of qubit states $|0\rangle$ and $|1\rangle$ to be determined by distinct circularly polarized lights $\sigma ^{-}$ and $\sigma ^{+}$, respectively. (**d**) The same polarization elements trap opposite qubit states in Rb. (**e**, **f**) For in-plane trapping, the 2D standing wave is formed by 831 nm linearly polarized light, trapping both Cs and Rb at the nodes and anti-nodes of the standing wave via blue- and red-detuned transitions. (**g**) Potential Energy Curve (PEC) with S- and P-wave scattering of the Rb atom’s Rydberg electron from a ground state Cs atom. Here the coupling of the Rydberg state $|57D_{5/2},5/2\rangle$ with the neighboring states $|55H+57D_{3/2}+58P_{\{1/2,3/2\}}+59S_{1/2}\rangle$ is considered under Eq. (10). The interaction strength is plotted along the radial direction with $\theta = \pi /2$, which is perpendicular to the lattice plane.

An alternative approach for sensing the logical qubit could be provided by Rydberg–Fermi interaction^52^. Here the interaction-induced level-shift occurs by Fermi scattering of the central atom’s Rydberg electron from the plaquette atoms^50–52^ in a qubit-dependent lattice^53–64^, see Figs. 4, 5, and 6. Depending on the state of the logical qubit, different numbers of plaquette atoms are spatially shifted inside or outside of the central atom’s Rydberg wave function. This would define the Fermi-scattering induced level-shift leading to controlled photon emission similar to the dipolar scheme explained in Fig. 1.

#### Rydberg–Fermi scheme

The level scheme of the central atoms for both four- and six-qubit encodings is similar to Fig. 1b,c, except that the plaquette driving is absent, as shown in Fig. 5b,d. The Rydberg laser, denoted as $\Omega _r$, excites the auxiliary atom at the center of the desired plaquette, as depicted in Fig. 6a. Applying a spin-dependent lattice shift perpendicular to the 2D lattice results in dual spin/spatial encoding of the plaquette qubits. By exciting the central atom to the $|57D_{5/2},5/2\rangle$ Rydberg state, the electron wave function overlaps exclusively with plaquette qubits in the spin state ${|{0}\rangle }$, as shown in Fig. 4f. The level scheme for the *four-qubit encoding* is illustrated in Fig. 4. In the case of ${|{1_L}\rangle }$, the arrangement of physical qubits in the qubit-dependent lattice positions two plaquette atoms within the Rydberg wave function of the central atom, making the $\Omega _r$ laser resonant with the shifted Rydberg level, $\delta _{\text {r}}({|{1_L}\rangle })=0$. For ${|{0_L}\rangle }$, the arrangement of the plaquette contains either $S_p=0$ or 4 atoms within the Rydberg wave function, resulting in an effective detuning of $\delta _{\text {r}}({|{0_L}\rangle })=2V_{\text {RF}}$, as described in Eq. (10).

In the case of *six-qubit encoding*^12^, where the logical basis consists of the odd and even parity states of $\bar{Z}=I Z I Z I Z$, exciting the Rydberg superposition state $R_{n,l}(r)(Y_2^2(\theta ,\phi )+Y_{2}^{-1}(\theta ,\phi ))/\sqrt{2}$ can realize the desired parity-photon entangling operation in a triangular lattice, as illustrated in Fig. 5c. Fermi scattering of the central atom’s Rydberg electron from every other site in a hexagonal plaquette structure is conditional on the physical qubits being in the ${|{0}\rangle }$ state. This interaction causes an effective level shift quantified by the spin number of the three atoms associated with non identity Pauli operators, as shown in Fig. 5d. The two-color $\Omega _r$ laser in Fig. 5d are tuned to be in resonance with the odd-parity state of the $\bar{Z}$ stabilizer.

#### Rydberg–Fermi interaction

The resulting *Rydberg–Fermi interaction* caused by the scattering of a Rydberg electron from a single neutral atom is quantified by^83–85^:

10 $$\begin{aligned} V_{\text {RF}}=(2\pi \frac{\tan (\delta ^s)}{k(R)}-6\pi \frac{\tan (\delta ^p)}{k^3(R)}{\mathop {\nabla }\limits ^{\leftarrow }}_{\textbf{r}}.{\mathop {\nabla }\limits ^{\rightarrow }}_{\textbf{r}})\delta (\textbf{r}-\textbf{R}) \end{aligned}$$

where **r** and **R** being the positions of the Rydberg electron and a ground state atom with respect to the ionic core, and $\delta ^{\{s,p\}}$ are the triplet s- and p-wave scattering phase shift of Rydberg electron from a neighboring ground state atom^86^. The coupling of the Rydberg state $|57D_{5/2},5/2\rangle$ with the neighboring states $|55H+57D_{3/2}+58P_{\{1/2,3/2\}}+59S_{1/2}\rangle$ is calculated under the interaction of Eq. (10) as a function of interatomic distance *R*, see Fig. 6g and Supplemental material. Here ${|{nH}\rangle }=\sum _{l,m} {|{n,l,m}\rangle }$ represents the Hydrogen state with semi degenerate orbital angular momentum numbers $2<l<n$. The matrix elements in the manifold of coupled states are given by:

11 $$\begin{aligned} H_{nlm,n'l'm'}(\textbf{R})= & \, {\langle {\psi _{nlm}(\textbf{R})}|} V_{\text {RF}}{|{\psi _{n'l'm'}(\textbf{R})}\rangle } \quad \quad \quad \quad \nonumber \\ H_{nlm,nlm}= & {} -\frac{Ry}{n^{*2}} \end{aligned}$$

where *Ry* is the Rydberg constant of *Cs* atoms and $n^{*}$ is the effective Rydberg principal number. By diagonalizing 8000 coupled states' Hamiltonian, the energy potential is plotted in Fig. 6g. In the optical-lattice discussed in Section “Qubit-dependent lattice”, the angle between counter-propagating trapping lasers is adjusted to obtain an interspecies lattice constant of $a=295$nm. The corresponding Fermi scattering of the Rydberg electron from the neighboring lattice site results in a 60 MHz level shift of the Rydberg state, ideal for fast quantum operations.

#### Qubit-dependent lattice

To avoid laser cross talk with logical qubits, a dual-species optical lattice of $^{133}$Cs and $^{87}$Rb is considered^87–89^. Trapping in the lattice plane is achieved using 831 nm linearly polarized light that is blue-detuned for $^{133}$Cs and red-detuned for $^{87}$Rb, resulting in traping at the nodes and antinodes of the standing wave, respectively (see Fig. 6e,f). The spin-dependent lattice shift along the *z*-axis is generated by 873 nm counter-propagating linearly polarized lights, as depicted in Fig. 6b.

With a relative shift between the fields’ polarizations $2\theta$, the total electric field can be expressed as the sum of right and left circularly polarized components: $E=E_0 \exp (-i \nu t)(\sigma ^{+}\sin (kz+\theta )+\sigma ^{-}\sin (kz-\theta ))$. To achieve a spin-dependent lattice shift, the spin polarizabilities must be linked to different circular polarization components of light^53–55^. In $^{133}$Cs, to cancel the polarizabilities of unwanted light elements (shown by dashed lines in Fig. 6c), the trapping laser is tuned between the $6P_{3/2}$ and $6P_{1/2}$ states, causing the AC Stark shifts of these two levels to cancel each other. Consequently, the $m_j=\pm 1/2$ levels of the ground state are trapped by potentials $V_{\pm }=\alpha |E_0|^2 \sin (kz\pm \theta )$, respectively. In $^{87}$Rb, the same fields are red-detuned from the $5P_{1/2}$ state, causing the same polarization components to trap opposite qubit states compared to $^{133}$Cs (see Fig. 6a,d).

The physical qubits are encoded in the Cs atoms placed on the plaquette while the central auxiliary atoms are Rb. This arrangement optimizes the interaction since electron scattering from Cs atoms features resonance at lower kinetic energies close to the last lobe^90^. The ${|{0}\rangle }$ and ${|{1}\rangle }$ qubit states of Cs are trapped in different $\sigma ^{-}$ and $\sigma ^{+}$ standing waves, respectively. For the electronic states of the central Rb atoms, we select ${|{s}\rangle }={|{5S_{1/2}, F=1,m_f=1}\rangle }$ and ${|{e}\rangle }={|{5S_{1/2}, F=2,m_f=-1}\rangle }$, which have the same distribution of magnetic quantum numbers $m_j$ and are therefore confined to the same qubit-dependent site. The chosen intermediate and Rydberg state are ${|{p}\rangle }={|{6P_{3/2}}\rangle }$ and ${|{r}\rangle }=|57D_{5/2},5/2\rangle$.

Single-site addressing has been achieved in dense lattices with interatomic distances of 537 nm^91^. Other approaches for single-site addressing include using local light shifts or deploying standing-wave two-photon driving techniques, as explained in the Supplementary material.

#### Preserving the ground motional state

Upon excitation of the central atom, the trapping potential experienced by the plaquette atoms in ${|{1_t}\rangle }$ Wannier states is modified by the Rydberg–Fermi potential. To prevent unwanted entanglement between computational and motional states, it is crucial to apply $\Omega _s$ adiabatically^52^. During the operation, the trap evolution experienced by plaquette atoms in ${|{1_t}\rangle }$ is given by $U_{trap}=U_{op}+P_{r_c}(t)V_{RF}$, where $P_{r_c}=(\frac{\Omega _s}{\Omega _r})^2$ represents the Rydberg population of the central atom, and $U_{op}$ is the optical trap potential. The Wannier state of ${|{1_t}\rangle }$ adapts continuously and stays close to the instantaneous ground motional state in an adiabatic operation, i.e. $\dot{\omega }_{trap}\ll \omega ^2_{trap}$^92^. The $\Omega _s$ Gaussian pulses with FWHM$>0.5\mu$s preserve the ground motional state. Additionally, applying quantum-twist optical lattices^93^ with ultra-tight confinement enables faster adiabatic operations.

#### Operation fidelity in Rydberg–Fermi scheme

The optimum realistic parameters for driving the central atom include $\Omega _r/2\pi = 38$MHz, max$(\Omega _s)/2\pi =10$MHz, $g/2\pi = 8.5$MHz, $\Gamma _c/2\pi =750$kHz^71^, $\Delta /2\pi =33$MHz and $\delta _{\text {r}}({|{0_L}\rangle })/2\pi =20$MHz. Considering a Gaussian pulse $\Omega _s(t)=\Omega _s \exp (-t^2/\sigma ^2)$ with $\sigma =3.5\mu$s, the operation fidelity of ${|{0}\rangle }({|{{0_L}}\rangle }+{|{{1_L}}\rangle })/\sqrt{2}\rightarrow ({|{\text {early}}\rangle }{|{0_L}\rangle }+{|{\text {late}}\rangle }{|{1_L}\rangle })/\sqrt{2}$ for the specified parameters, under the numerical simulation, is Fid = 99.6%.

### Supplementary Information

Supplementary Information.

## Data Availability

All data needed to evaluate the conclusions in the article are presented in the article and the Supplementary. Additional data related to this paper may be requested from the corresponding author.

## Code availability

Codes could be provided upon reasonable request.
