# Supplementary material for: Universal terminal for cloud quantum computing
Source: Sci Rep. 2024 Jul 4;14:15412. doi: 10.1038/s41598-024-65899-0 (PMC11224409; doi:10.1038/s41598-024-65899-0)
Supplement: Supplementary file 1 — Supplementary Information. [file 41598_2024_65899_MOESM1_ESM.pdf]

SUPPLEMENTAL DOCUMENT: UNIVERSAL TERMINAL FOR CLOUD QUANTUM COMPUTING

S1. CLOSING THE RYDBERG-MOLECULE DECOHERENCE CHANNEL

In a Bose-Einstein condensate (BEC), the decoherence rate can be significantly increased by the Fermi scattering of a Rydberg electron from a free ground state atom [1]. This decoherence arises due to the attractive Rydberg-Fermi potential near the ionic core, which causes the two interacting atoms to approach a very small separation of approximately 2 nm. At this distance, the binding energy of the molecules can ionize the Rydberg electron, resulting in the formation of a Cs_2^+ molecule [2].

When atoms are confined in a lattice, the interatomic separation can be tuned to correspond to the last lobe of the Rydberg-Fermi interaction featuring restoring force, thus maintaining the desired interatomic distance. In this configuration, without mass transport, the step-wise decay or ionization of the Rydberg atom is unlikely. This is because the Rydberg-Fermi binding energy at 170nm lattice constant used in this paper is orders of magnitude smaller than the closest Rydberg levels for the principal numbers applied here. The ion-pair formation is also highly unlikely in this system [2].

S2. PONDEROMOTIVE FORCE

The Rydberg wave-function is spread over a large area which experience the variation of the standing wave. As a result, the ponderomotive force perturbs the electron wave-function and affects the Fermi scattering. In a standing wave described by$E(x, y, t) = 2E_0 \cos(kx)\cos(ky)\cos(\omega t)$, the ponderomotive force $F_p = -\frac{e^2 \nabla |E|^2}{4m_e\omega^2}$ [3] pushes the electron towards the nodes of the standing wave.

In the proposed dual species lattice depicted in Fig. 5e,f, Cs and Rb atoms experience blue- and red-detuned light, respectively, causing them to be localized at the nodes and anti-nodes of the standing wave. As a result, the ponderomotive force pushes the electron away from the ionic core and towards the plaquette atoms, enhancing the interaction. Additionally, the ponderomotive force creates a four-leaf clover pattern in the lattice plane, further confining the electron cloud around the plaquette atoms. strengthening the Fermi scattering. Notably, the ponderomotive potential maintains the symmetry of the electron cloud at the positions of the plaquette atoms, ensuring that the enhanced interaction is uniform and stable.

S3. SINGLE SITE ADDRESSING

Laser cross-talk and misalignment can affect the accuracy of the Rydberg-Fermi scheme, especially when operating in compact lattices. To address this, two approaches for single-sitetransition are discussed. The first approach utilizes local light-shift, while the second employs standing wave driving. In both methods, only the targeted auxiliary atom is transferred to the ground hyperfine state $|s\rangle$, while the remaining atoms stay in a different hyperfine ground state $|g\rangle$, which is not affected by the rest of the operation.

Applying local light-shift

Single-site addressing can be performed by applying site-selective differential light-shift to the $|s\rangle\langle g|$ transition [4]. By focusing a 788nm auxiliary laser on the targeted site, only the desired atom is brought into resonance with the microwave or optical Raman transition.

The efficiency of this method has been quantified for cases where the 788nm light is focused to $1/e^2$ intensity waists of $w = 500$ nm or 370 nm using a NA=0.68 microscope [4]. The single-site alignment accuracy of 25 nm, as used in [4], can be further improved through techniques such as sub-wavelength localization of atoms [5, 6].

In this setup, the generated light-shift by the focused laser is given by $U_{\rm LS}(x, y) = U_{\rm LS} \exp\left(-2\frac{(x-x_0)^2+(y-y_0)^2}{w^2}\right)$, with $r_0 = \sqrt{x_0^2 + y_0^2}$ representing the laser misalignment. At the central site, this misalignment causes a detuning $\Delta(r_0) = U_{\rm LS}(1 - e^{-2r_0^2/w_0^2})$ in the $|s\rangle\langle g|$ transition, altering the effective Rabi frequency to $\tilde{\Omega} = \sqrt{\Omega^2 + \Delta^2}$. This results in imperfect transition over the π pulse duration $\tau = \frac{\pi}{\Omega}$, introducing an error of $E_a = \frac{\Omega^2}{\tilde{\Omega}^2}\sin^2(\tilde{\Omega}\tau/2)$.

Considering the uncertainty in addressing a specific site within distance r_0, the alignment error must be averaged as $\bar{E}_a = \frac{1}{\pi r_0^2}\int_0^{r_0} E_a(r) 2\pi r\, \mathrm{d}r$. Accounting for the error distribution profile, the optimal operating time is modified from $\tau = \frac{\pi}{\Omega}$ to $\tau_{\mathrm{opt}} = \frac{\pi}{\sqrt{\Omega^2+\Delta(\frac{3}{4}r_0)}}$. The averaged error of the central atom $\bar{E}_a$ is plotted by dashed lines in Fig. S1 as a function of U_{LS}/Ω. Infidelities caused by laser misalignment can be suppressed through spatial beam shaping [7].

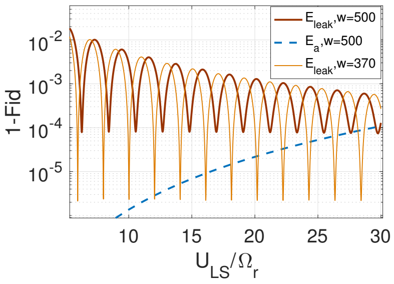

FIG. S1. Single-site addressing with local light-shift– To achieve single-site initialization in $|s\rangle$, the 788nm laser is focused to a $1/e^2$ intensity waist of w =500nm and 370nm with an alignment accuracy of $r_0 = 25$nm, generating a differential light-shift U_{LS} on the $|s\rangle\langle g|$ transition. Consequently, only the targeted site is brought into resonance with the microwave or optical Raman transition between the two hyperfine ground states $|s\rangle$ and $|g\rangle$. The errors are averaged over the laser's misalignment area, represented as a circle with radius r_0, and also over the Wannier state of atoms, which is considered as a Gaussian with a half-width diameter of 30 nm. The solid line indicates the leakage error of neighboring auxiliary atoms ($\bar{E}_{\mathrm{leak}}$), while the dashed line represents the rotation error of the targeted site due to laser misalignment ($\bar{E}_a$). The oscillations arise from changes in the effective Rabi frequency ($\tilde{\Omega}$) at the positions of neighboring auxiliary atoms. Fine-tuning the parameters ensures that neighboring sites remain in the $|s\rangle$ state.

Regarding laser cross-talk, the detuning experienced by neighboring atoms must be sufficiently large to prevent population leakage from the $|g\rangle$ state. In the proposed dual-species lattice, the nearest neighboring site with the same type of Rb atoms is separated by $2a$.

Over the operation time τ_{opt}, the leakage error is given by $E_{leak} = \frac{\Omega^2}{\Omega^2+\Delta(|\mathbf{r}|)^2}\sin^2(\frac{\Omega^2+\Delta(|\mathbf{r}|)^2}{\Omega^2+\Delta(\frac{3}{4}r_0)^2}\pi)$ where $|\mathbf{r}| = |\mathbf{a}-\mathbf{r_l}-\mathbf{r_n}|$ is the distance from the center of the laser beam to the neighboring auxiliary atom, and $\mathbf{2a}$ is the distance vector between the target and a neighboring auxiliary site. The reported leakage error, $\bar{E}_{\mathrm{leak}}$, is averaged over the laser misalignment $\mathbf{r}_l$ and the Wannier wavefunction of the atoms.

The variation of the leakage error is plotted by solid lines in Fig. S1 as a function of U_{LS}/Ω. Oscillations occur due to changes in the effective Rabi frequency $\tilde{\Omega}$ at the position of the neighboring Rb atom, leading to different leakage values after the π pulse. At large U_{LS}/Ω and also for weak laser focusing (large w), the variation in detuning over the plaquette atoms' wavefunctions is substantial, thus averaging the error over $\mathbf{r}_l$ and $\mathbf{r}_n$ smooths the oscillation pattern.

Considering both the leakage error $\bar{E}_{\mathrm{leak}}$ and the alignment error $\bar{E}_a$, Fig. S1 demonstrates that single-site operations with high fidelities are achievable in the designed setups discussed in the main text.

Interferometric approach

High-precision addressing of single lattice sites can be achieved using standing wave driving of a Raman transition, similar to the sub-wavelength localization approach described in references [5, 6, 8–10]. In this process, the ground state $|g\rangle$ of the desired site is exclusively initialized to $|s\rangle$ via an intermediate level $|6P\rangle$. The Λ transition, depicted in Fig. S2, contains a standing-wave driving field (Ω_c) and a focused laser (Ω_p).

The counter-propagating fields $\Omega_{c1q}\exp(ikq)$ and $\Omega_{c2q}\exp(-ikq+\phi_q)$, where $q \in \{x, y\}$, create the standing wave. The transition is governed by the dark state local STIRAP (Stimulated Raman Adiabatic Passage) mechanism. The dark state has spatially varying amplitudes, expressed as:

$$|D(\mathbf{r})\rangle = \frac{1}{\sqrt{\Omega_c(\mathbf{r})^2 + \Omega_p(\mathbf{r})^2}}[\Omega_c(\mathbf{r})|g\rangle - \Omega_p(\mathbf{r})|s\rangle]. \quad (1)$$

The operation steps are as follows:

1. Apply the Ω_{c1q} field.

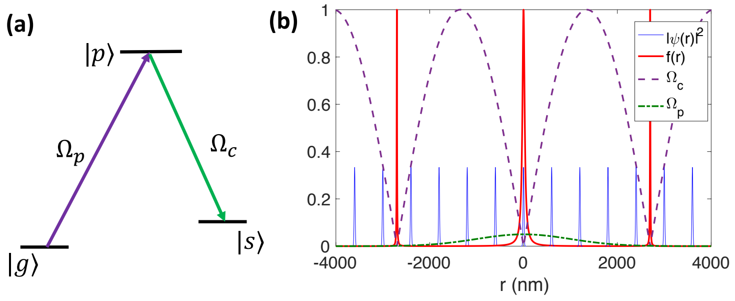

FIG. S2. **Single-Site Addressing with Standing-Wave Driving** – (a) the level scheme illustrates a Λ configuration for transferring the population from $|g\rangle$ to $|s\rangle$ at the nodes of the Ω_c standing-wave, where $\Omega_c \ll \Omega_p$. (b) The spatial profile of the transition Rabi frequencies Ω_c and Ω_p, the $|s\rangle\langle g|$ transition probabilities $f(x)$, and the spatial probability amplitude $|\psi(x)|^2$ of the atoms in $|g\rangle$ qubit states are plotted. The function $f(r)$ maps $|\psi(x)|^2$ to the $|s\rangle$ state upon the spatial overlap ,which is designed to occur exclusively at the targeted site. (b) The applied parameters are $\Omega_c/\Omega_p = 30$, $\tilde{k} = 1.2\mu m^{-1}$ and the Gaussian width of the Ω_p laser is $w = 2\mu$m.

2. Shine the probe field with a wide Gaussian profile, $\Omega_p(\mathbf{r}) = \Omega_p \exp\left(-\frac{(\mathbf{r}-\mathbf{r_0})^2}{w^2}\right)$, onto the targeted site, ensuring that $\Omega_p \ll \Omega_c$.
3. Adiabatically raise the Ω_{c2q} field [6], forming the desired standing wave. Adjust the ϕ_{2q} phase to position a node of the Ω_c standing wave at the location of the targeted site.
At locations away from the targeted node where $\Omega_p(\mathbf{r}) \ll \Omega_c(\mathbf{r})$, the dark state remains in the ground state $|g\rangle$. Conversely, at the designated node where $\Omega_p(\mathbf{r}) \gg \Omega_c(\mathbf{r})$, the dark state is predominantly composed of $|s\rangle$.

Taking into account the spatial probability distribution of ground-state atoms in the $|g\rangle$ state, $|\psi(\mathbf{r})|^2$, the local population of $|s\rangle$ after applying $\Omega_{p,c}$ fields can be expressed as $f(\mathbf{r})|\psi(\mathbf{r})|^2$, where $f(\mathbf{r})$ is derived from Eq. 1 as

$$f(r) = \frac{\Omega_p^2 \mathrm{e}^{-\frac{2(\mathbf{r}-\mathbf{r_0})^2}{w^2}}}{\Omega_p^2 \mathrm{e}^{-\frac{2(\mathbf{r}-\mathbf{r_0})^2}{w^2}} + \Omega_c^2 \sin^2 \tilde{k}(x-x_0) \sin^2 \tilde{k}(y-y_0)}, \tag{2}$$

where $\tilde{k} = k \sin\theta/2$ and θ is the angle between the Ω_{c1} and Ω_{c2} lasers. The narrow peaks of $f(r)$ at the nodes of Ω_c are depicted in Figure S2b. Away from the focusing point of Ω_p at $\mathbf{r_0}$, the width of $f(r)$ narrows and eventually disappears.

The full width at half maximum (FWHM) of an f peak located at r' is given by $\text{FWHM}_{f(r')} = 2\Omega_p \exp(-(\mathbf{r'} - \mathbf{r_0})^2/w^2)/\tilde{k}\Omega_c$ [5, 6]. The nearest peaks of f shown in Fig. S2b do not overlap with the atomic lattice sites. The farther peaks of f occur where the amplitude of Ω_p approaches zero.

In the final step, both the Ω_p and Ω_c lasers are ramped down simultaneously, preserving the ratio of $\Omega_c(t)/\Omega_p(t)$ to keep the dark state components unaltered. As a result, only the targeted site would be in the $|s\rangle$ state and hence experiences the level scheme described in Fig.1b,c of the main text.

The calibration of the Ω_c standing-wave nodes with the lattice sites can be performed using fluorescence imaging, as detailed in [6]. The standing-wave nodes are then precisely adjusted by fine-tuning the phase of the Ω_{c2q} field [11].

For the chosen parameters in Fig. S2b, where Rb atoms are separated by 600 nm in the lattice with an atomic confinement of FWHM = 20 nm, and the Ω_p laser is focused to a Gaussian width of 2 μm, the single-site addressing infidelity averaged over the qubit configurations is estimated to be 0.01. This calculation accounts for both the imperfect transition at the targeted site and the population leakage to neighboring sites.

S.4 CALCULATING THE RYDBERG-FERMI INTERACTION

For the p-wave scattering of Rydberg electron from the neighboring ground state atom, the gradient of the Rydberg wave-function $\psi = R_{nl}(r)Y_l^m(\theta,\phi)$ at the position of the neighboring lattice site is required which is

$$\nabla\psi(r,\theta,\phi) = \begin{bmatrix} \frac{\partial R_{nl}}{\partial r} Y_l^m \\ \frac{1}{r} R_{nl} \frac{\partial Y_l^m}{\partial \theta} \\ \frac{1}{r\sin\theta} R_{nl} \frac{\partial Y_l^m}{\partial \phi} \end{bmatrix} = \tag{3}$$

$$\begin{bmatrix} \frac{\partial R_{nl}(r)}{\partial r} Y_l^m(\theta,\phi) \\ \frac{1}{r} R_{nl}(r) \frac{1}{2}\sqrt{l^2-m^2}[Y_l^{m+1}(\theta,\phi)e^{-\mathrm{i}\phi} - (l+m+1)Y_l^{m-1}(\theta,\phi)e^{\mathrm{i}\phi}] \\ \mathrm{i}m\frac{\psi(r,\theta,\phi)}{r\sin(\theta)} \end{bmatrix}$$

in the spherical coordinate. The radial wave function and its derivative are calculated numerically using the Numerov technique. [12].

[1] J. B. Balewski, et al., *Nature* **502**, 664 (2013).

[2] T. Niederprüm, O. Thomas, T. Manthey, T. M. Weber, and H. Ott, *Phys. Rev. Lett.* **115**, 013003 (2015).

[3] Anderson, S., Raithel, G. Ionization of Rydberg atoms by standing-wave light fields. Nat Commun **4**, 2967 (2013).

[4] Weitenberg, Christof, et al. "Single-spin addressing in an atomic Mott insulator." *Nature* **471** 319 (2011).

[5] Miles, J. A., Z. J. Simmons, and D. D. Yavuz. "Subwavelength localization of atomic excitation using electromagnetically induced transparency." *Phys. Rev. X* **3** 031014, (2013)

[6] Subhankar, Sarthak, Yang Wang, Tsz-Chun Tsui, S. L. Rolston, and James V. Porto. "Nanoscale atomic density microscopy." *Phys. Rev. X* **9**, 021002 (2019).

[7] Gillen-Christandl, K., Gillen, G. D., Piotrowicz, M. J., and Saffman, M. Comparison of Gaussian and super Gaussian laser beams for addressing atomic qubits. *Appl. Phys. B* **122**, 1 (2016).

[8] Agarwal, Girish S., and Kishore T. Kapale. "Subwavelength atom localization via coherent population trapping." *J Phys B-At Mol Opt* **39**, 3437 (2006).

[9] J. Cho, Addressing individual atoms in optical lattices with standing-wave driving fields, *Phys. Rev. Lett.* **99**, 020502 (2007)

[10] K. T. Kapale and G. S. Agarwal, Subnanoscale resolution for microscopy via coherent population trapping, *Opt. Lett.* **35**, 2792, (2010).

[11] Teleshevsky, V. I., S. V. Bushuev, and S. G. Grishin. "Acousto-optic method of electronic laser phase control for laser interferometry." *Journal of Physics: Conference Series. IOP Publishing* **1546**, 1 (2020).

[12] T. Gallagher, *Rydberg Atoms* No. 3. Cambridge University Press, (2005).
